# Creating a Theoretically Grounded Gaming App to Increase Adherence to Pre-Exposure Prophylaxis: Lessons From the Development of the Viral Combat Mobile Phone Game

**DOI:** 10.2196/11861

**Published:** 2019-03-27

**Authors:** Laura Whiteley, Leandro Mena, Lacey K Craker, Meredith Garver Healy, Larry K Brown

**Affiliations:** 1 Department of Psychiatry Warren Alpert Medical School Brown University Providence, RI United States; 2 University of Mississippi Medical Center University of Mississippi Jackson, MS United States; 3 Young Adult Behavioral Health Department of Psychiatry and Human Behavior Rhode Island Hospital Providence, RI United States

**Keywords:** cell phone, HIV, young adult, sexual and gender minorities

## Abstract

**Background:**

In the United States, young minority men who have sex with men (MSM) are most likely to become infected with HIV. The use of antiretroviral medications to reduce the risk of acquiring HIV infection (pre-exposure prophylaxis, PrEP) is an efficacious and promising prevention strategy. There have been significant advances regarding PrEP, including the definitive demonstration that PrEP reduces HIV acquisition and the development of clinical prescribing guidelines. Despite these promising events, the practical implementation of PrEP can be challenging. Data show that PrEP’s safety and effectiveness could be greatly compromised by suboptimal adherence to treatment, and there is concern about the potential for an increase in HIV risk behavior among PrEP users. Due to these challenges, the prescribing of PrEP should be accompanied by behavioral interventions to promote adherence.

**Objective:**

This study aimed to develop an immersive, action-oriented iPhone gaming intervention to improve motivation for adherence to PrEP.

**Methods:**

Game development was guided by social learning theory, taking into consideration the perspectives of young adult MSM who are taking PrEP. A total of 20 young men who have sex with men (YMSM; aged 18-35 years) were recruited from a sexually transmitted infection (STI), HIV testing, and PrEP care clinic in Jackson, Mississippi, between October 2016 and June 2017. They participated in qualitative interviews guided by the information-motivation-behavioral skills (IMB) model of behavior change. The mean age of participants was 26 years, and all the participants identified as male. Acceptability of the game was assessed with the Client Service Questionnaire and session evaluation form.

**Results:**

A number of themes emerged that informed game development. YMSM taking PrEP desired informational game content that included new and comprehensive details about the effectiveness of PrEP, details about PrEP as it relates to doctors’ visits, and general information about STIs other than HIV. Motivational themes that emerged were the desire for enhancement of future orientation; reinforcement of positive influences from partners, parents, and friends; collaboration with health care providers; decreasing stigma; and a focus on personal relevance of PrEP-related medical care. Behavioral skills themes centered around self-efficacy and strategies for adherence to PrEP and self-care.

**Conclusions:**

We utilized youth feedback, IMB, and agile software development to create a multilevel, immersive, action-oriented iPhone gaming intervention to improve motivation for adherence to PrEP. There is a dearth of gaming interventions for persons on PrEP. This study is a significant step in working toward the development and testing of an iPhone gaming intervention to decrease HIV risk and promote adherence to PrEP for YMSM.

## Introduction

### Background

The primary prevention of HIV infection remains a crucial priority. In 2016, there were 39,782 new HIV diagnoses in the United States, and 67% of these new diagnoses were of gay and bisexual men [1]. HIV also disproportionately affects communities of color. Despite only comprising 12.6% of the US population, nearly 45% of all new HIV infections occur among African Americans. The use of antiretroviral medications to reduce the risk of acquiring HIV infection (pre-exposure prophylaxis, PrEP) is an efficacious and promising new prevention strategy [1,2]. There have been significant advances regarding PrEP including the definitive demonstration that PrEP reduces HIV acquisition. Despite promising data, the practical implementation of PrEP is challenging [3-10]. Unfortunately, individuals who are often at the highest risk of HIV infection and eligible for PrEP come from populations that historically have been underserved by health care [11,12]. Therefore, engaging diverse persons on PrEP in care is challenging, and reinforcement and support for patients and doctors is required [11,13-15]. Behavioral Interventions promoting adherence to comprehensive PrEP treatment and healthy sexual behaviors will need to be tailored to diverse, underserved, and at-risk populations and will need to reinforce the clinician-patient relationship.

In addition, the behavioral interventions accompanying PrEP need to be scalable, cost-effective, and easily integrated into clinical settings [2,16]. Without these necessary components, integration of behavioral interventions into clinical settings cannot be realistically sustained. Utilizing gaming technology to deliver behavioral interventions for young men who have sex with men (YMSM) can improve intrinsic motivation and information and build behavioral skills for adherence. The use of an intervention that utilizes gaming technology is particularly compelling for use with younger adults, as this age group is at the highest risk of acquiring HIV and this age group has most actively inquired about PrEP in clinical settings. In addition, 72% of young male adults in the United States say they play video games often or sometimes [17]. Gaming technology is also popular among minority men who have sex with men (MSM), the subgroup most at risk for acquiring HIV. In the past, gaming was mistakenly identified as a primarily adolescent and heterosexually dominated activity, but current data support that gaming is actually quite diverse [18]. MSM gamers, referred to as *gaymers* in pop culture and mainstream articles, are highly represented on the web and increasingly shape the market [19-21]. In addition, within the United States, African Americans aged 18 to 35 years represent the most active and fastest growing user group of mobile phones [22,23], and more than half of the reported video gaming occurs on portable devices [18,21,22]. The widespread appeal and use of mobile phones and video game playing creates a unique opportunity to deliver health education to diverse YMSM on PrEP during leisure time, outside of the clinic, and in a manner that is cost-effective and easily scalable [24-27].

Interactive game play has been shown to enhance players’ motivation to improve health behaviors and self-care in a variety of clinical settings and populations. Games can attract and maintain attention, a key component for effective behavior change. Compelling interactive phone-based games can expose players to essential health-related content thousands of times and also give players unlimited opportunities to rehearse new skills and receive personalized feedback on health choices made within the game [28-30]. Games have been shown to be efficacious in promoting fitness, improving weight management, and improving safer sex skills [27,28,30,31]. For example, an HIV and AIDS prevention computer game called Life Challenge was developed by the New York State Department of Health to enhance safer sex negotiation by adolescents and young adults. The game showed significant improvement in self-efficacy for partner negotiation and condom skills for those who started with the least self-efficacy [30]. Two pregnancy prevention games, The Baby Game and Romance, designed for sexually active young adults, showed trends in improving knowledge and attitudes about parenting and unprotected sexual behaviors [29]. Video games have also been applied to improve self-management skills and healthy behaviors in those living with asthma, diabetes, and cancer [32-36]. For example, a video game named Re-Mission (tested with adolescents and young adults aged 13-29 years) was designed as an action-adventure in which the main character shoots cancer-causing agents in the bloodstream. In a randomized control study, 375 male and female participants who played Re-Mission had significantly improved adherence to trimethoprim-sulfamethoxazole (TMP/SMX; *P*=.01) and 6-mercaptopurine (*P*=.002) after an average of only 10.7 hours of play. Adherence to TMP/SMX by those playing Re-Mission was 19% greater than those in the control group. Self-efficacy (*P*=.01) and knowledge (*P*=.03) also increased [35,36]. Thus, appealing interactive games can target information, motivation, and skills for medical care and have led to a broad spectrum of desirable health outcomes including increases in knowledge, attitude changes, and increased medication adherence [27,31,37-39].

### Gaps in Literature

Despite the promise and popularity of digital games, there is a paucity of interventions or publications related to gaming for persons on PrEP [40]. Relatedly, there are games in development for YMSM either living with HIV or at risk for HIV [41-45]. For example, our research group has developed a gaming intervention to improve antiretroviral treatment (ART) adherence for youth and young adults living with HIV that informs the development of this game [40]. LeGrand et al have published a description of the development phase of a game entitled Epic Allies. Epic Allies is designed to improve ART uptake, engagement in care, and adherence among HIV positive YMSM and transgender women who have sex with men. While playing Epic Allies, users can earn medals and tokens for taking medications and reading health-related studies. [42]. Another intervention called PlayForward aims to reduce the risk for HIV among at-risk, ethnic, and racial minority adolescents. This tablet-based game provides an interactive world using an avatar where players face challenges such as peer pressure to drink alcohol or engage in other risky sexual behaviors [43]. A mobile phone–optimized intervention entitled healthMpowerment is designed to reduce sexual risk behaviors among YMSM. In this intervention, YMSM can acquire reputation points through reading information about HIV, playing sexually transmitted infection (STI)–related games, and through positive interactions with other users. Despite the promise and popularity of digital games, there are no published or presented abstracts related to gaming for persons on PrEP that we could find.

### This Study

We developed a gaming intervention to improve adherence to PrEP for YMSM aged 18 to 35 years. This intervention integrates a smart pill bottle cap (that measures adherence) with an iPhone game. The gaming intervention was informed by the information-motivation-behavioral skills (IMB) theory of learning. The IMB model, consistent with social learning theory, is broadly applicable and can be used to guide game development and create theoretically consistent gaming content [46-50]. The iPhone gaming app was designed for participants to experience absorbing action-oriented adventures that increase information about their health (eg, knowledge about PrEP and HIV prevention and adherence), improve motivation (eg, action figures experience health benefits of adherence to PrEP), and build skills (action figures interact with clinicians at appointments, take medications as prescribed, and practice safe sex; see Multimedia Appendices 1-13). Adherence (measured by the smart pill bottle cap) and game-related text messages are integrated into the gaming intervention. Multiple reviews have demonstrated that behavioral interventions shown to be most efficacious are those tailored for the target population and preceded by formative research to inform intervention development [51,52]. The aim of this study was to describe the development of this iPhone gaming app entitled ViralCombat.

## Methods

### Gaming App Development

Development of ViralCombat was accomplished using iterative and collaborative procedures to integrate the clinical experiences of YMSM taking PrEP, academic researchers, and technology partners. Game development was guided by qualitative interviews with a group of MSM aged between 18 and 35 years taking PrEP. Guided by the principles of agile software development [53], the qualitative interviews and the game and app programming were synergistic. Agile software development aims for continuous design testing and adaptation based on continuous feedback [53].

### Sample and Recruitment

Males aged between 18 and 35 years were eligible for enrollment in the study according to the following criteria: (1) English-speaking, (2) currently taking PrEP, (3) reported having sex with other men, and (4) able to give consent and not impaired by cognitive or medical limitations as per clinical assessment. Those who did not meet the abovementioned inclusion criteria were excluded. We recruited 20 YMSM for qualitative interviews to guide game development after institutional review board’s (IRB) approval. Subjects were from a convenience sample recruited from a PrEP clinic in Jackson, Mississippi, between October 2016 and June 2017. Subjects were approached by research staff with an IRB-approved flyer, and written consent was obtained upon meeting with study staff for the qualitative interview. Overall, 20 subjects were approached over the course of the interviews, and all of them consented and completed the interview. Subjects were recruited until data saturation was achieved, and a relative balance in the sample was achieved based on age greater than 26 years versus younger. The mean age of participants was 26 years (range: 18-35 years; 11 out of the 20 were younger than 26 years). A majority of the participants (17/20, 85%) identified as African American, 100% (20/20) identified as MSM, 70% (13/20) completed 12th grade, 75% (15/20) had been taking PrEP longer than 6 months, 75% (15/20) reported missing a dose of PrEP in the previous week, 45% (9/20) reported missing 3 doses or more in the previous week, and 75% (5/20) had been taking PrEP for 6 months or longer.

### Procedures

The study was approved by the hospital’s IRB, and participant consent and interviews were conducted in a private room located in a PrEP clinic in Jackson, Mississippi. Interviews were conducted by 1 of the 2 MDs (psychiatrists) with support from a trained research assistant, and subjects were reimbursed US $50 per survey (for a total of US $150) for their time. The psychiatrists who conducted the interviews did not provide medical or clinical services in the HIV clinic. Interviews lasted between 45 and 60 min and were digitally recorded. As we adapted our gaming intervention from games that were already developed (Dr. Nano X and BattleViro), the system and the framework (eg, code, database, and design) were already in place at the beginning of the project. Adaptations to the game occurred as themes emerged from the interviews [41]. Biweekly meetings were held with the programmers to discuss all adaptations, including changes to content, game graphics framework, and game messaging.

### Adaptation of the Information-Motivation-Behavioral Skills Adherence Gaming Intervention

The IMB gaming app for persons on PrEP, entitled ViralCombat, was adapted from the popular Mission Critical Studios game entitled Dr. Nano X as well as our previously developed game for persons on ART entitled BattleViro [40,41]. Dr. Nano X is a 5-star-rated mobile game (the highest rating possible) in the iTunes app store and is available on both Android and iPhone. The ART adherence game, named BattleViro, has been previously described in the *Journal of Medical Internet Research* [41]. We worked directly with the development team at Mission Critical Studios to develop ViralCombat using Dr. Nano X and BattleViro as a basic framework. Adapting our game to promote PrEP adherence from already existing games greatly decreased the cost of the project. Characters, actions, and IMB messaging about PrEP were built specifically for ViralCombat; however, we were able to reuse backgrounds, mechanisms of game play and controls, and many sound effects from Dr. Nano X and BattleViro [54,55]. ViralCombat game levels are distinct from those in BattleViro, and they include organ systems such as the penis, anus, and mouth.

We worked iteratively with youth living with HIV and Mission Critical Studios to create the app. The game starts with a 45-second narrative movie that explains the storyline and game objectives. The player is told by the narrator, in a deep and dramatic voice, “you have been chosen, due to your smart decisions and healthy choices, to be cloned and shrunken in order to enter your own body to destroy attacking viruses and infections.” The narrator emphasizes that “in order to acquire ammunition and strength, you must take you medication in real life and also pick up pills during play.” Players are given a tutorial on game actions and can also design their individual and diverse characters. As players successfully battle HIV, engage with providers, and take medication, they move to new, distinctive levels (arterial system, penis, anus, and mouth). Messages from the doctors, nurses, and friends encourage and provide clues during difficult twists and turns in the battle. Answering quiz questions from clinician avatars allows each player to earn strength and points; wrong answers are corrected and explained. Players find medication, strength, and points by acting on positive suggestions. During each mission, the player’s score (pill count and health) is shown. All character control and gaming is done by touch screen technology on the phone; no additional accessories are needed for play. Throughout the game, the terms “HIV” and “PrEP” are seen on the screen, but they are never part of an audio feature, so stigmatizing information cannot be overheard by another person. The game-related text messages have gaming graphics, similar to other apps and games, and the app uses push notifications to engage gamers in play. If players were less than 90% adherent during the week, phrases such as “Missing you in Combat” and “Get back in the game” are texted to their phones. Congratulatory short message service (SMS) text messages such as “Great job in battle” and “You are fighting off virus well” are sent for greater than 90% adherence (see Multimedia Appendix 2). Throughout gaming, the mission stays the same: kill the virus and build strength through taking medicine, learning information, improving motivation, and engaging with healthy characters to improve adherence to PrEP and build HIV preventative skills.

### Interview Topics

Interviews with YMSM taking PrEP-guided game development were conducted. A total of 11 participants were shown the storyboard of the game and completed interviews. When the iPhone version became available, 9 participants were given the game on an iPhone, and their feedback was elicited through an interview. Therefore, qualitative feedback was received from all 20 participants. The interview guide consisted of focused but open-ended questions aimed at maximizing participant responses (see Textbox 1). Participants were asked about information relevant to medication adherence, motivating factors to adherence, and behavioral skills needed for adherence. The IMB model guided interview content, and participants were also queried about their general gaming experience and their reactions to the storyboard and gaming content.

### Information Needed for Adherence

Participants were asked about knowledge and information that influences their adherence to PrEP and engagement in medical appointments. Questions included:

What type of information from doctors or friends makes it easier to take PrEP?

What information makes it easier to come to PrEP related appointments?

This part of the interview aimed to understand the specific knowledge about HIV and PrEP that promotes adherence behaviors. For example, some probes focused on how side effects and other health-related information can influence adherence to medication and care (for more examples, see Textbox 1).

### Motivation for Adherence

Participants were queried about motivational issues related to adherence to PrEP with probes such as:

I would like to hear about what you think the serious issues are surrounding PrEP and taking medications to prevent HIV,

What are the things that make it hard to take PrEP?

This part of the interview was dedicated to understanding both personal and social motivations for adherence to PrEP. Queries were focused on the positive and negative attitudes toward taking PrEP, perceived negative effects of nonadherence to PrEP, and the individual’s perceptions of social support from significant others, family, friends, and medical care providers (for more examples, see Textbox 1).

### Behavioral Skills for Adherence

Participants were also asked about their ability to perform necessary adherence and HIV preventative tasks and their perceived self-efficacy for these tasks. Questions included:

What are the ways that you stay safe from HIV?

What are the ways that you remember to take PrEP and remember your PrEP related appointments?

What events in your life make it harder to remember to take PrEP? Or remember your appointments?

**Qualitative interview guide based on the information-motivation-behavioral (IMB) skills model.**

Information

- What knowledge or information about HIV and pre-exposure prophylaxis (PrEP) is helpful to know?
- What knowledge or information helps you to take PrEP daily?
- Does knowing about side effects change decision making around taking PrEP?

Motivation

- What are the main issues in coming for PrEP related medical appointments?
- What are the things that make it hard to take PrEP?
- What are the attitudes or feelings that people like you have that make it harder to take PrEP? Or easier to take PrEP?
- How do partners, your family, and your community play a role in adherence to PrEP related care?

Behavioral skills

- Do you use alarms, your phone, or reminders to remember to take PrEP?
- What do you do if you miss a dose of PrEP?
- What are the strategies for adherence to PrEP over time and across different situations?
- Are there things that you do such as eating, or avoiding certain substances, that make taking PrEP easier?

General gaming attitudes

- What is your reaction to getting some PrEP related and HIV related information and skills in a game?
- Do you ever play games that teach you facts or in which you learn something?
- Do you go online or use your phone to learn information about your health?
- Have you ever played a health-related game before on your phone or at a computer?

Reactions to ViralCombat

- What did you like and not like about the game?
- What do you think this activity is trying to teach you?
- How much did the material look like the other games that you play?
- How could this activity or content be improved for others like you?
- Now that you have seen this game, would you want to play it?
- Before you came here today, did you ever find anything like this on a game on a phone or on a computer?
- Would you be worried about playing the game when others could see it?
- What would you say if someone asked you about the game?

We also asked participants about strategies for self-reinforcement for adherence over time and across different situations. We asked questions such as:

Do you consciously think about your PrEP medication schedule on a long-term basis?

What strategies have you used or developed to remember to take PrEP or to go to your appointments based on your activities?

This part of the interview aimed to assess perceived abilities and strategies to store, obtain, and self-cue the use of medications despite challenges and across situations (for more examples, see Textbox 1).

### General Gaming Attitudes

Participants were also asked about their general attitudes and experiences with games. Participants were asked questions such as:

What games do you, or people you know, play on the cellphone?

What types of graphics, avatars, and rewards do you like? And what do you not like?

How are games useful? Do you develop any skills when you play games?

These queries elicited descriptions of popular game activities and attitudes about gaming. The responses were used to make the format and game mechanics of ViralCombat engaging and immersive (for more examples, see Textbox 1).

### ViralCombat Storyboard and iPhone Game

Participants were asked for feedback about the storyboard or the iPhone game (once the mobile game was ready) with the probes such as:

What was the main point of this activity?

What could you learn from this activity?

Would you recommend this type of game to your friends?

What is your reaction to having some HIV and PrEP related information and skills in an iPhone game?

After the first version of the game was developed on the iPhone, participants were asked additional and modified probes such as:

Is the game easy to navigate and easy to understand?

Did any part of the game not work?

Are there other topics that the game should cover that it does not?

Answers to these questions guided the iterative development of the game levels, actions, characters, and graphics (for more examples, see Textbox 1).

### Medication Adherence Monitoring Tracking and Game-Related Text Messages

Participants were also asked about the electronic pill monitoring organizers and adherence-related text messages. We queried participants about the smart pill bottle cap that can electronically monitor, measure, and securely relay adherence pill bottle openings to our research team. Participants were asked about the use of reminder messages with a game-related graphic. Feedback was elicited about game-related messages if players missed a dose using texted phrases such as “Missing you in Combat” and “Get in the game.” Feedback was also received about SMS text messages if doses are taken on time such as “Great job in battle” and “You are fighting off virus well!” (see Multimedia Appendix 2).

### Quantitative Feasibility and Acceptability Data

The game was developed iteratively. A total of 11 participants were shown the storyboard of the game for approximately 30 min; qualitative feedback was elicited, and game development on the iPhone occurred. After the development of the iPhone version of ViralCombat, 9 of the 20 participants played the game for 45 to 50 min with the interviewer in the room (see Multimedia Appendices 1-13). These 9 participants were shown each of the game levels on the phone by the interviewer and played each level. After playing the game, these 9 participants completed qualitative interviews, and then, written or quantitative feedback was obtained. Quantitative feedback was collected from these 9 participants using versions of the Client Service Questionnaire (CSQ) and the session evaluation form (SEF) [56,57]. Both these instruments were developed to measure client satisfaction and perspectives on intervention aspects. The CSQ consists of 8 items that assess general satisfaction with the game. An example query from the CSQ is “In an overall, general sense, how satisfied are you with the game?” (for which the response options are 4=“Very satisfied”; 3=“Mostly satisfied”; 2=“Indifferent or mildly dissatisfied”; and 1=“Quite dissatisfied”) [56]. We did not calculate an alpha coefficient for this sample because of the small number of participants completing the CSQ (n=9). In other studies, the internal consistency of the CSQ-8 is high, with alpha coefficients ranging from .84 to .93 for the CSQ [58]. The SEF contains 13 items that assess the feasibility and perceived utility of activities in the game. For example, the SEF states: “I will be able to apply what I learned from this game in my life” (for which the response options are 1=“Strongly agree”; 2=“Agree”; 3=“Disagree”; and 4=“Strongly disagree”) [57]. As the SEF asks about specific the utility of separate elements of the intervention, and not about singular constructs, alpha coefficients for the SEF are not commonly calculated.

### Data Analysis

#### Qualitative Data

Trained research assistants transcribed verbatim the digital audio recordings of each interview. Then, the MD- or PhD-level research team member reviewed the transcripts with the digital recording for accuracy. Qualitative data analysis followed the tenets of thematic analysis, which consisted of sequential steps [59,60], and interviews continued until data saturation was achieved. The research team familiarized themselves with the data, reviewing each transcription. Next, the research team met weekly and generated a list of codes as they emerged. The team generated a thematic table of the analyses and checked the extent to which the emerging themes reflected the coded data [59,61]. The team grouped the themes under the general categories of the interview guide (ART information, ART motivation, ART behavioral skills, general game attitudes, and reactions to ViralCombat). Themes were examined in their relation to perceived utility of the game and for factors that would improve or detract from the game’s impact. Team discussion and interviews continued until discrepancies were resolved.

#### Quantitative Data

Participant responses on the CSQ and SEF were entered into an Excel file, and responses were verified with a second entry. Categorical response frequencies were calculated for each item of both scales. General acceptability of the intervention is illustrated using individual items from the scales. CSQ items are reported using the proportion of participants endorsing satisfaction with the intervention (response options “Very satisfied” and “Mostly satisfied” were combined). SEF items are reported using the proportion endorsing “agreement” with feasibility and utility of the game (response options “Strongly agree” and “Agree” were combined).

## Results

### Reactions to ViralCombat Storyboard and iPhone Game

Interviews from both the storyboard and iPhone game revealed a number of themes that guided game development. Participants desired informational game content that included new and comprehensive details about PrEP, details about PrEP as it relates to doctors’ visits, and general health information. Motivational themes that emerged were the desire for enhancement of future orientation; reinforcement of positive influences from peers, partners, and friends; collaboration with health care providers; decreasing stigma; and increasing personal relevance of HIV prevention. Behavioral skills themes centered around self-efficacy and strategies for PrEP adherence and medical care (see Textbox 2 for qualitative interview themes and resulting game adaptations based on the IMB model).

Textbox 2 highlights the barriers and facilitators to adherence expressed by our participants and the corresponding gaming app action or message that was adapted to enhance facilitators or challenge barriers. Textbox 2 also includes general gaming attitudes that influenced the development of ViralCombat and specific reactions to the ViralCombat storyboard and iPhone game. In addition to the themes in Textbox 2, participants who played the game on the phone said that important gaming characteristics included directly destroying and fighting off HIV in game play, earning health points by taking pills that looked like PrEP, a prologue or introduction with a dramatic voice-over, and images about HIV and STIs that were relevant. Participants wanted levels that become increasingly difficult (for a sense of accomplishment). Participants did not want HIV or PrEP in the title of the game because of concerns about privacy and stigma but wanted to fight graphics labeled “HIV” and wanted to see the word PrEP during gaming. A 29-year-old black male participant said:

I like that the organ systems are the penis and the anus, it’s funny, but also realistic. It helped me learn about how infection with HIV happens.

A 30-year-old white male participant said:

It was awesome to shoot HIV virus before it enters the body, and I like that HIV looked like it would under a microscope.

An 18-year-old black male stated:

I liked taking pills that look exactly like PrEP; it’s like my real-life.

A 21-year-old white male stated (see Textbox 2):

It was cool that I am playing a game about preventing, that it was like tailored to me and my friends.

I think my other friends who think about HIV would like playing this.

The music and sound is really awesome.

The game was iteratively changed as comments were received that indicated a need for alteration. For example, facts about HIV prevention and the benefits of adherence to PrEP were made more sophisticated when multiple participants gave feedback such that they knew most of the information given in the game and that they wanted more detailed information about side effects. Many participants also asked for information about substance use. A representative comment was from a 19-year-old black male who said:

I think there should be facts in the game about other health stuff, about smoking and drinking on PrEP.

Many participants also wanted more guidance through the levels. For example, a 29-year-old white male participant stated:

I would like better hints on each level on how to beat the level.

An 18-year-old black male said (see Textbox 2):

Clues or hints when it gets hard would make this better.

### Monitoring Pill Bottle Opening and Game-Related Text Messages

We asked participants about the SMS text messages with gaming graphics and the use of a smart pill cap that measured adherence. During the interviews, we demonstrated how openings of the pill bottle were measured wirelessly, and we showed participants sample adherence-informed SMS text messages. When looking at the smart pill cap, an 18-year-old black male participant stated:

It’s cool how it links with game.

It’s awesome that there is a bottle that knows what you are doing.

The feedback about the cap was extremely positive; however, a 19-year-old black male participant stated:

The pill cap is OK, I don’t love how big it is. People can see it in my bookbag more.

A 26-year-old white male described:

It would be better if it was a bit smaller, but I like that it can record when I take pills, that helps me.

Although 2 participants had negative remarks about the size of the pill bottle, 18 out of 20 participants responded they liked the cap design and did not think the size of the cap was an impediment to use. During interviews, participants were also shown SMS text messages that corresponded to adherence data from the smart pill cap. Participants liked the proposed SMS text messages, and an 18-year-old black male described:

These texts cue me to take my meds.

A 30-year-old black male stated:

I like the game pictures.

...the texts made me smile and also I felt like they were just for me.

A 35-year-old black male participant described:

The texts got me in the mood to play the game again.

Of the participants, 3 described that they wanted more variation in the game-related texts:

Seeing the same graphics again and again is boring.

35-year-old black male and a 19-year-old black male

An 18-year-old black male stated:

I would like more variety in the game texts.

**Qualitative interview themes and resulting game adaptations based on the information-motivation-behavioral skills (IMB) model.**

Information

- New and comprehensive details about pre-exposure prophylaxis (PrEP) and how PrEP prevents HIV
  - Game includes complex and realistic information about PrEP. Participants fight off HIV in each organ. HIV is graphically represented. Facts about HIV and PrEP are imparted at every level. HIV is pictured.
- PrEP as it relates to doctors’ visits
  - Terms and verbiage often used at PrEP-related doctor visits are used and defined in the game frequently. The importance of regular HIV testing and testing for other STIs is explained.
- General health information
  - Participants learn about sexually transmitted infections that PrEP does not protect them from getting (gonorrhea, syphilis, herpes, genital warts, and human papillomavirus).
  - Participants in the game receive messages about how exercise and healthy eating also affect health. Participants also receive messages about avoiding illicit substances and how illicit substances can increase risk behavior.

Motivation

- Enhancement of future orientation
  - Messages about staying healthy for family, friends, and children scroll through game. As gaming participant takes more pills and builds more health, they are able to move through levels, receive more artillery, and have more success staying healthy.
- Personal relevance of HIV
  - Participants are shrunken down to enter into their own body to fight HIV. During game play, participants see how PrEP works to prevent HIV infection.
- Collaborating with health care providers
  - Throughout the game, participants partner with doctors to advance to the next level, build strength, and collect artillery.
- Reinforce influences from peers, partners, and friends
  - Scrolling messages remind gamers that staying healthy for partners, friends, and family is meaningful for themselves and for the loved ones in their lives.
- Decrease stigma
  - Participants are empowered to kill HIV before it enters the body and gain points with each healthy decision.
  - Adherence to PrEP is valued as healthy and smart decision-making.

Behavioral skills

- Self-efficacy for PrEP adherence and PrEP-related medical care
  - Solving problems and collecting pills or swallowing pills in the game leads to more points, which leads to more strength, more health, and more artillery. This leads to more game play. Perseverance throughout levels leads to success in the game.
- Strategies for medication adherence and self-care
  - Scrolling messages encourage the participant to use PrEP, schedule routine doctors’ appointments, and ask providers or doctors questions about topics relevant to HIV prevention.

General gaming attitudes

- Desire for games with levels, sound effects, and colorful graphics. Ability to earn points in the game and choose avatars
  - Levels or organ systems become increasingly difficult (for a sense of accomplishment). Background music, sound effects, and dramatic voice-overs are included. Colorful graphics are included and change often. Choice of avatars is available. Participants earn points in the game by swallowing pills.

Reactions to ViralCombat

- Desire for game action that is realistic with relevant information about PrEP and HIV prevention.
  - Participants can directly destroy HIV in game play, and graphics look like PrEP and HIV.
  - Participants improve health and gain points in the game by taking virtual PrEP pills.
  - Participants liked progression through organ systems, with information about PrEP and HIV that is pertinent to that organ system.

### Acceptability and Feasibility

CSQ and SEF scores were available from participants who played the game on the iPhone for 45 to 50 min. The responses were generally quite positive, 88% (8/9) of the participants were satisfied with the activities in the game, 77% (7/9) learned a lot from this game, 88% (8/9) thought the game was well organized, 77% (7/9) felt game topics were interesting, 100% (9/9) felt they would recommend the game to a friend, 77% (7/9) felt game topics stimulated their interest in the material, 100% (9/9) felt that game topics were relevant to their lives, and 66% (6/9) felt they were able to do the activities in the game (see Table 1). The gaming intervention was improved based on the above acceptability and feasibility feedback from the CSQ and SEF and also on the feedback from the qualitative interviews (see Textbox 2). Specifically, game play was made easier with written messages and hints throughout each level on how to move forward. We also improved narrated instructions at the beginning of each level to assist players. General health facts about smoking, side effects of PrEP, and how substances such as drugs and alcohol can increase risky behavior were incorporated into the game to increase perceived relevance. We also incorporated more detailed information about HIV and how PrEP works to improve learning and interest. We also included a condom quiz game and information about other STIs (see Multimedia Appendices 7, 8, and 11). To improve SMS text messages, we included emojis and designed 5 different game-related SMS text messages utilizing a larger variety of game graphics based on participant feedback.

**Table 1:** iPhone game acceptability and feasibility scores (N=9).

Questionnaire or form		Participants endorsing statement, n (%)
**Client Service Questionnaire**		
	How would you rate the quality of the game?	8 (88)^a^
	Did you get desired information from the game?	6 (66)^a^
	To what extent does the game’s content meet your needs?	7 (77)^a^
	If a friend were interested in a similar program, would you recommend our game to him or her?	9 (100)^a^
	How satisfied are you with the amount of information you have received in the game?	7 (77)^a^
	Has the information you received helped you to deal more effectively with issues important to you?	8 (88)^a^
	In an overall, general sense, how satisfied are you with the game?	8 (88)^a^
	Would you come back to the game again?	9 (100)^a^
**Session evaluation form**		
	I learned a lot from the game.	7 (77)^b^
	I will be able to apply what I learned from the game in my life.	6 (66)^b^
	I was able to do the activities in the game.	8 (88)^b^
	The game was well organized.	7 (77)^b^
	The topic of the game was interesting.	7 (77)^b^
	The presentation of the information stimulated my interest in the material.	9 (100)^b^
	The topics of the game were relevant to my life.	8 (88)^b^
	The game was enjoyable.	8 (88)^b^
	I would recommend the game to others.	9 (100)^b^
	I felt comfortable during game play.	9 (100)^b^

^a^Proportion of participants that endorsed “Very satisfied” and “Mostly satisfied.”

^b^Proportion of participants that endorsed “Strongly agree” and “Agree.”

## Discussion

### Principal Findings

In this project, we utilized in-depth interviewing, focused by social learning theory (IMB), to create an iPhone gaming intervention to measure and improve treatment PrEP adherence and decrease HIV risk for YMSM [46-48,53,62]. A number of themes emerged through qualitative interviews with young men that informed game development. We found that YMSM desired informational game content that included comprehensive details about how HIV is transmitted, PrEP-related doctors’ visits, side effects of PrEP, and general health information. Motivational themes or findings that emerged were the desire for enhancement of future orientation; the need for reinforcement of positive influences from peers, partners, and friends; and the promotion of collaboration with health care providers. Motivational themes also included decreasing stigma and increasing personal relevance of HIV prevention. Behavioral skills themes or findings centered around self-efficacy and strategies for PrEP medication and appointment adherence including regular HIV and STI testing.

Using the IMB theory in the development of this game ensured that the intervention was informed by decades of prevention research. This study demonstrates that qualitative assessment, social learning theory, and agile software development can complement each other and are important components to the development of a tailored and clinically relevant app. Participant data were used throughout the development of the game and informed the informational, motivational, and behavioral skill-building components of the game. Using a storyboard provided the research team with opportunities to share concept models with participants early on in the design process and gather feedback with respect to necessary modifications. Sharing the iPhone game with participants as it was developed also allowed for necessary, incremental improvements. YMSM at risk of HIV infection provided key qualitative insights with respect to the content and design and process of the game. Tailored games that are informed by those who will use them have more potential for effective integration and uptake in clinical settings [41].

Although iPhone games are pervasive in popular culture, no other gaming apps have been developed for YMSM on PrEP. Findings of this study highlight several important barriers and facilitators to PREP adherence for YMSM. Mobile interventions have the potential to reinforce skills learned in the clinic and require fewer resources to deliver patient-centered, evidence-based interventions [63]. Furthermore, apps and mobile phone games have the potential to engage YMSM in interventions, who otherwise may not be willing or able to participate in intervention programs.

Gaming and mobile apps also have the potential to advance the delivery of information and promote healthy decision-making in disproportionately affected populations, including minority men who often have less access to medical care and support [60]. National data from the Pew Research Center indicate that younger, ethnic and racial minority populations use mobile phones frequently [64]. The adolescents and young adults in this study repeatedly expressed having access to, and familiarity with, iPhones. This widespread use of iPhones facilitates the uptake of gaming apps in clinical populations. Therefore, mobile technologies such as mobile phone games and apps have a great potential to enhance medical care and prevention interventions for populations who are disproportionately affected by HIV and other STIs.

### Limitations

Findings should be interpreted in light of study limitations. First, our participants were recruited from a PrEP clinic in Jackson, Mississippi. This clinic may not be representative of all PrEP clinics in the United States or internationally. Therefore, the generalizability of the data collected to inform the development of the app is unknown and may be limited. Second, this study focused on individual PrEP user perspectives. It may be equally important to integrate the perspectives of clinicians, friends, or partners into the game. In the future, including friends and social networks into gaming prevention programs could be novel and effective. Perspectives of partners, friends, and clinicians could also lead to a more robust understanding of barriers and facilitators to adherence to PrEP and PrEP-related medical care. Therefore, future research could examine the utility of integrating feedback from clinicians and friends into the gaming app. Finally, this app was developed for the iPhone. Development of the app for Android devices could allow for greater availability of the game and could be a forthcoming step in the future phases of research.

### Conclusions

This study is a significant step in the development and testing of an iPhone gaming app to promote adherence to PrEP and PrEP-related medical care. The long-term goal of this research program is to test ViralCombat in a randomized trial and examine if the game is effective in changing PrEP-related attitudes and adherence-related behaviors. If the results appear promising, the research team can distribute the technology procedures for the intervention to relevant and interested clinics, community-based organizations, the Centers for AIDS Research, and AIDS Trials Networks. The app could also be made available on iTunes and Google Play (Android) for a nominal fee.

There are many advantages to using newer interactive technology to improve adherence rather than traditional face-to-face counseling, including scalability, efficiency, and cost-effectiveness. As electronic games are highly appealing to young men [25], they are a natural opportunity to deliver health education during leisure time and outside of the clinic [25-27,52]. Games can attract and maintain attention, which is a key component for effective behavior change. Compelling interactive games can expose players to essential health-related content thousands of times and also give players unlimited opportunities to rehearse new skills and receive personalized feedback on health choices made within the game [28,35]. We are not aware of other adherence interventions that integrate medication adherence monitoring technology, SMS text messaging, and a theoretically informed game to improve information, motivation, and behavioral skills for PrEP adherence. An intervention with these components may empower and engage YMSM, aid clinics, and result in improvements in health.

## Appendix

Multimedia Appendix 1 ViralCombat main menu graphic.

Multimedia Appendix 2 Example of a short message service text message to the participants.

Multimedia Appendix 3 ViralCombat game menu.

Multimedia Appendix 4 Short narrative movie at the beginning of the game.

Multimedia Appendix 5 Players can design and individualize their game character.

Multimedia Appendix 6 Players are shrunken down to be able to enter the body to fight off HIV.

Multimedia Appendix 7 Answering questions with allied doctors and building knowledge help each player successfully move to the next level or area of the body. Example of questions answered incorrectly.

Multimedia Appendix 8 Answering questions with allied doctors and building knowledge help each player successfully move to the next level or area of the body. Example of questions answered correctly.

Multimedia Appendix 9 Example of a player gaining strength in the anus level by collecting health pills and condoms.

Multimedia Appendix 10 As players travel through the bloodstream, they must fight off viruses and gain health pills.

Multimedia Appendix 11 Throughout ViralCombat, players engage in various games in order to move on to the next level. In the Condom Sequence game, players must go through the correct sequence of steps for correctly using a condom.

Multimedia Appendix 12 Players prevent HIV and other sexually transmitted infections in the penis by killing viruses and other bacteria.

Multimedia Appendix 13 Summary of points earned at the end of each level.

## Abbreviations

ART: antiretroviral treatment
CSQ: Client Service Questionnaire
IMB: information-motivation-behavioral skills
IRB: institutional review board
MSM: men who have sex with men
PrEP: pre-exposure prophylaxis
SEF: session evaluation form
SMS: short message service
STI: sexually transmitted infection
TMP/SMX: trimethoprim-sulfamethoxazole
YMSM: young men who have sex with men
