# Supplementary figures and images for: Creating a Theoretically Grounded Gaming App to Increase Adherence to Pre-Exposure Prophylaxis: Lessons From the Development of the Viral Combat Mobile Phone Game
Source: JMIR Serious Games. 2019 Mar 27;7(1):e11861. doi: 10.2196/11861 (PMC6456850; doi:10.2196/11861)

Multimedia Appendix 1. Viral Combat main menu graphic.

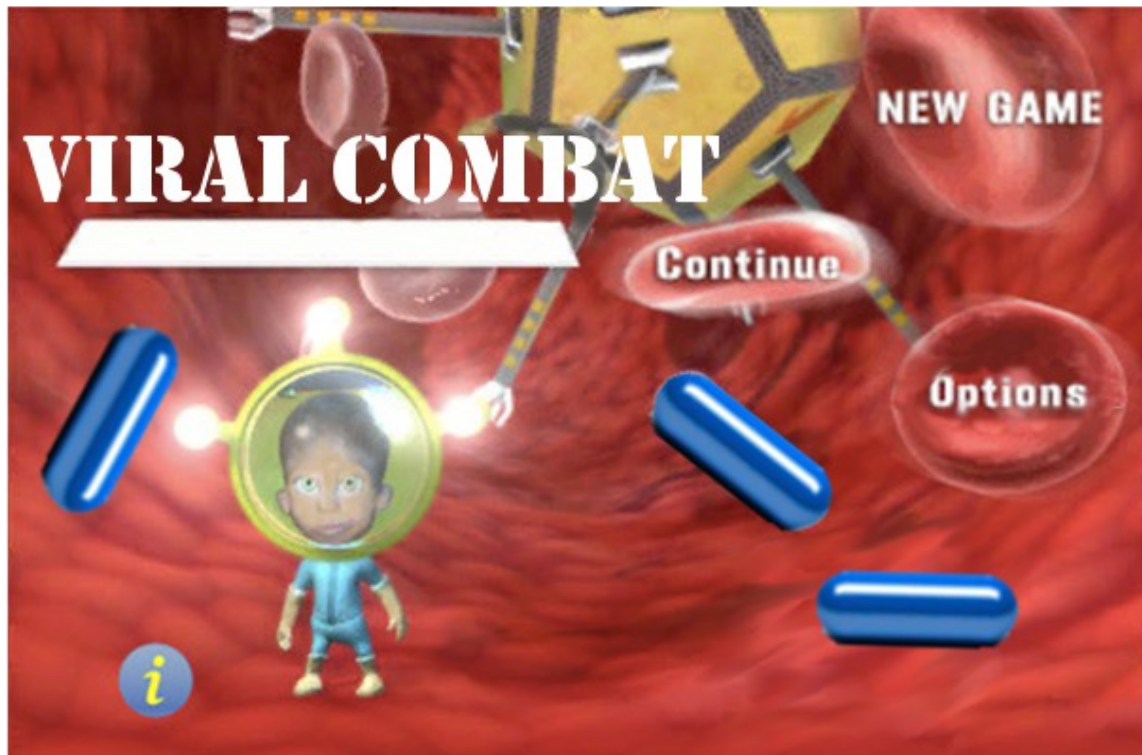

Supplement: Multimedia Appendix 1 [file games_v7i1e11861_app1.pdf]

Multimedia Appendix 2. Example of an SMS text message to the participants.

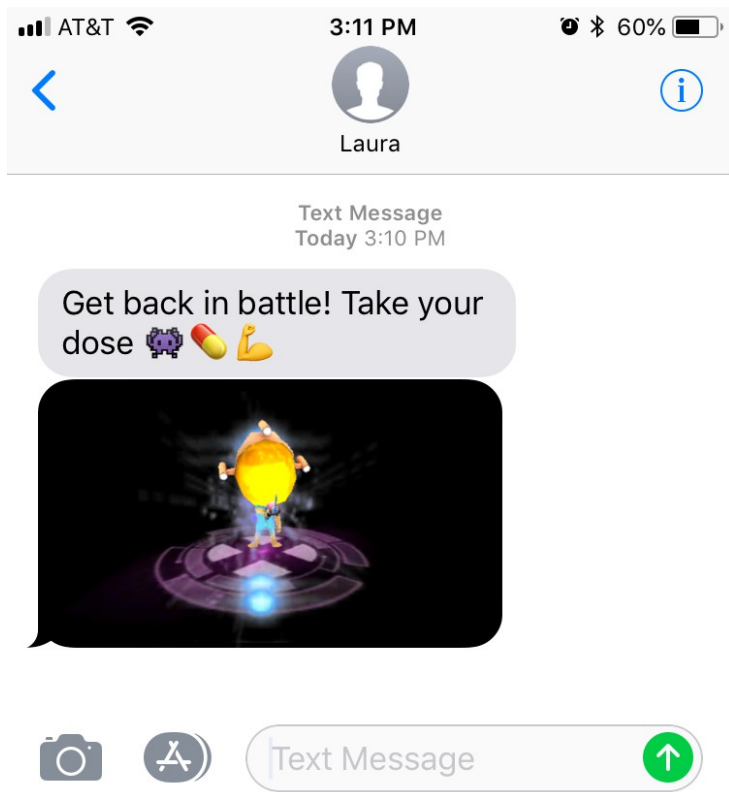

Supplement: Multimedia Appendix 2 [file games_v7i1e11861_app2.pdf]

Multimedia Appendix 3. Viral Combat game menu.

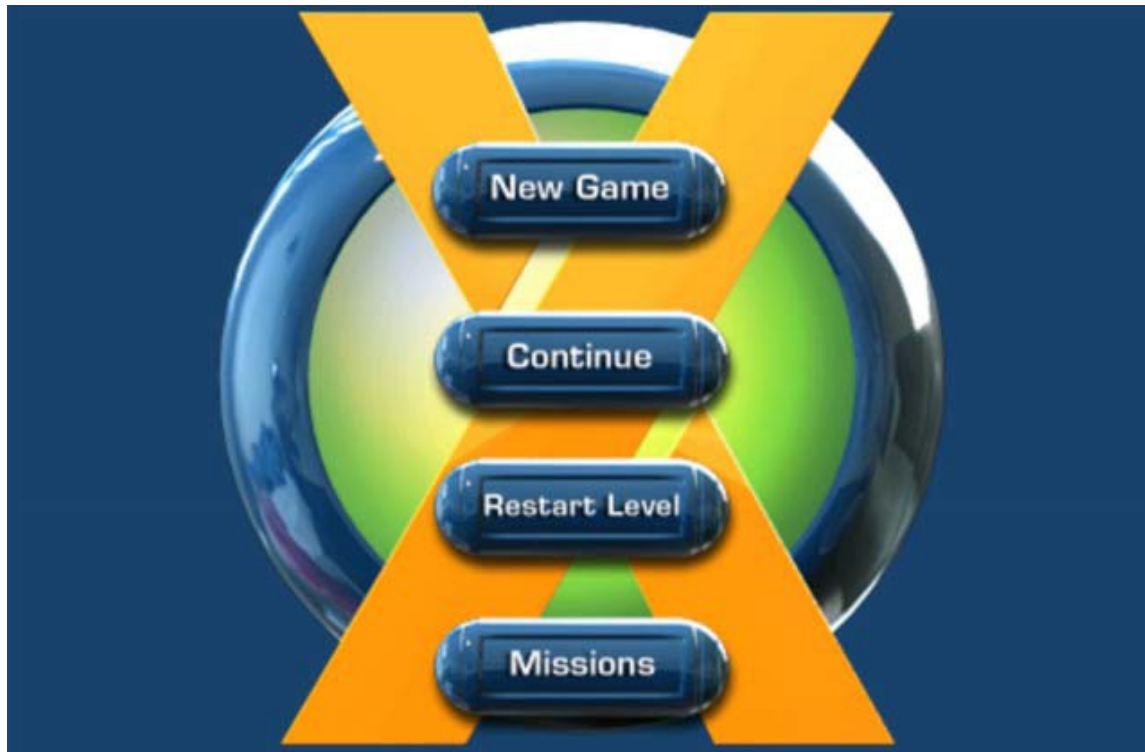

Supplement: Multimedia Appendix 3 [file games_v7i1e11861_app3.pdf]

Multimedia Appendix 4. Short narrative movie at the beginning of the game.

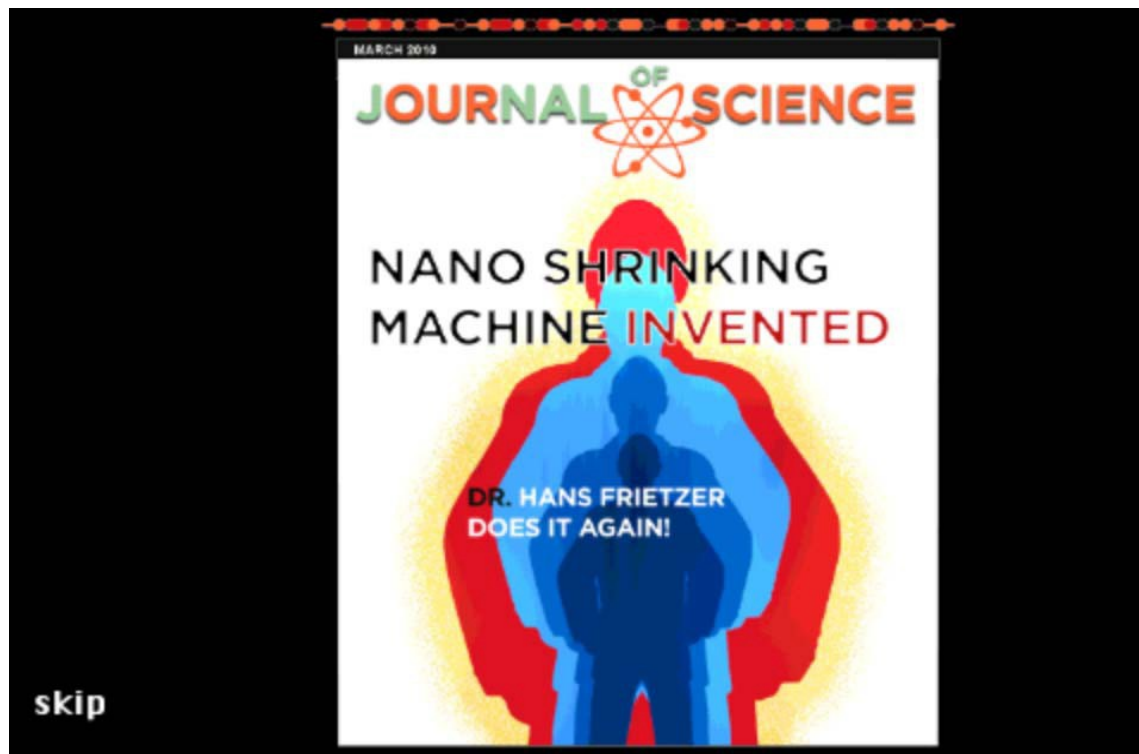

Supplement: Multimedia Appendix 4 [file games_v7i1e11861_app4.pdf]

Multimedia Appendix 5. Players can design and individualize their game character.

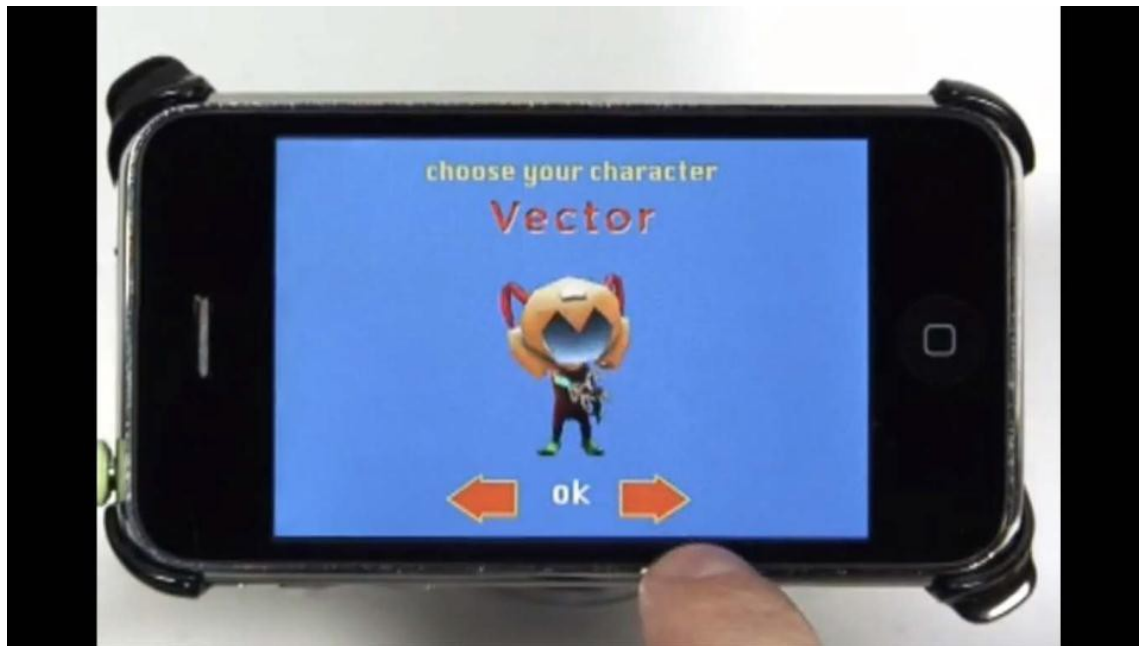

Supplement: Multimedia Appendix 5 [file games_v7i1e11861_app5.pdf]

Multimedia Appendix 6. Players are shrunk down to be able to enter the body to fight off HIV.

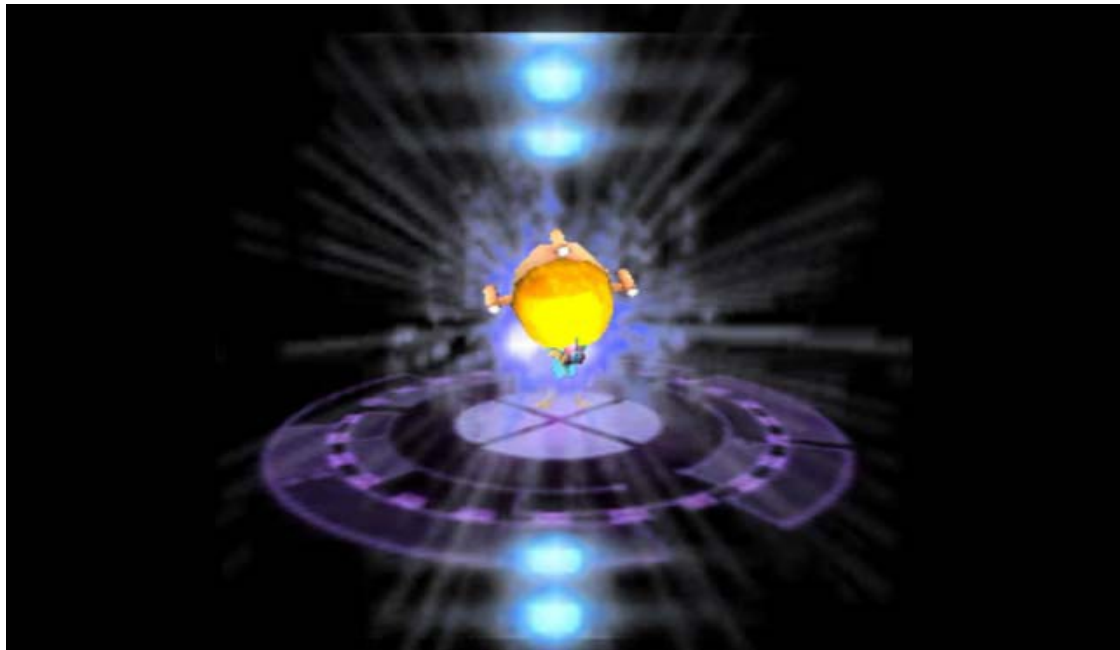

Supplement: Multimedia Appendix 6 [file games_v7i1e11861_app6.pdf]

Multimedia Appendix 13. Summary of points earned at the end of each level.

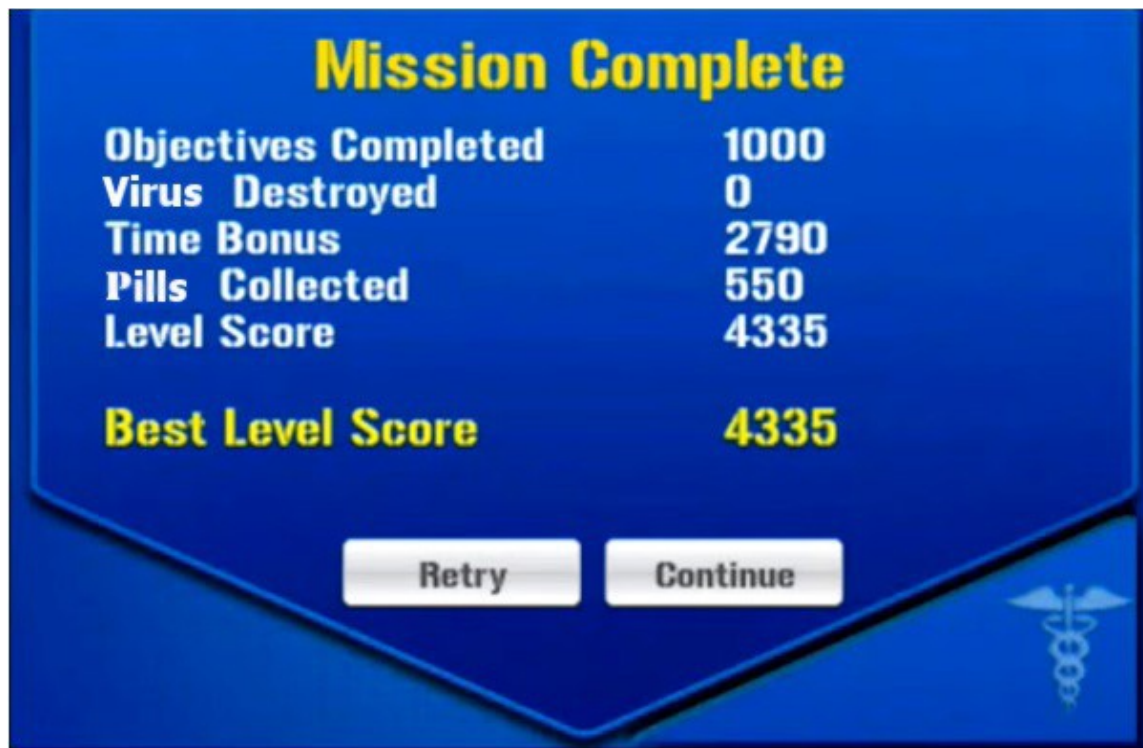

Supplement: Multimedia Appendix 13 [file games_v7i1e11861_app13.pdf]
