# Supplementary material for: Creating a Theoretically Grounded Gaming App to Increase Adherence to Pre-Exposure Prophylaxis: Lessons From the Development of the Viral Combat Mobile Phone Game
Source: JMIR Serious Games. 2019 Mar 27;7(1):e11861. doi: 10.2196/11861 (PMC6456850; doi:10.2196/11861)

Multimedia Appendix 8. Answering questions with allied doctors, and building knowledge, helps each player successfully move to the next level or area of the body. Example of question answered correctly.

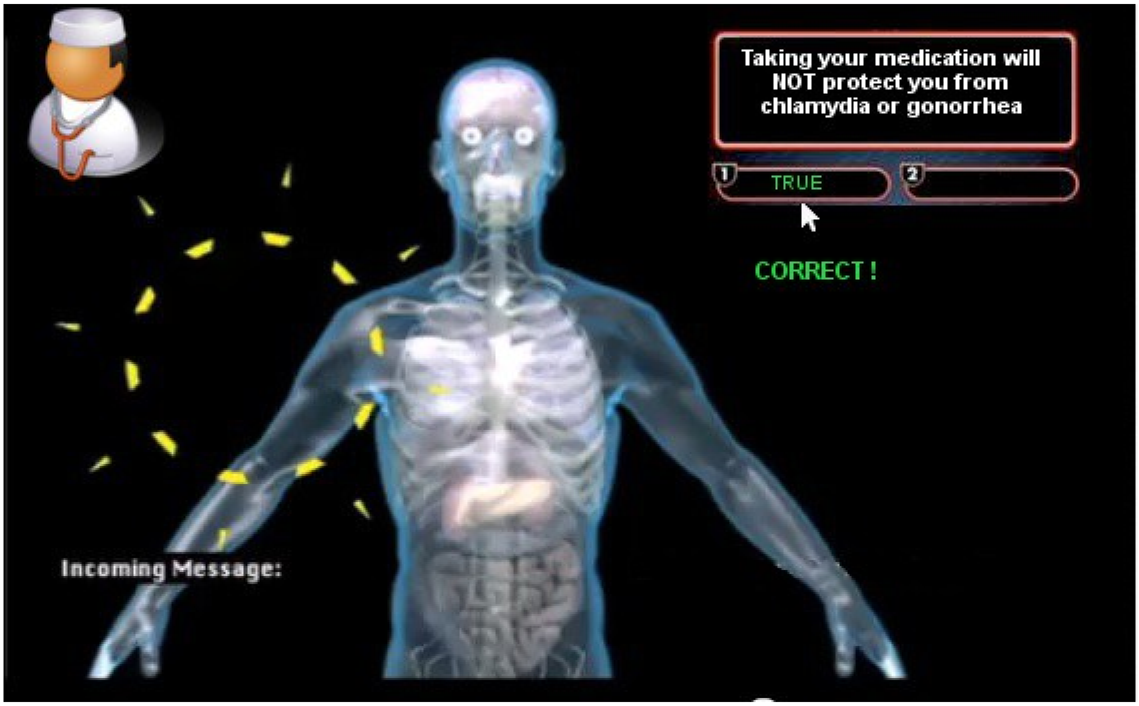

Supplement: Multimedia Appendix 8 [file games_v7i1e11861_app8.pdf]
