# Supplementary material for: Creating a Theoretically Grounded Gaming App to Increase Adherence to Pre-Exposure Prophylaxis: Lessons From the Development of the Viral Combat Mobile Phone Game
Source: JMIR Serious Games. 2019 Mar 27;7(1):e11861. doi: 10.2196/11861 (PMC6456850; doi:10.2196/11861)

Multimedia Appendix 9. Example of a player gaining strength in the anus level by collecting health pills and condoms.

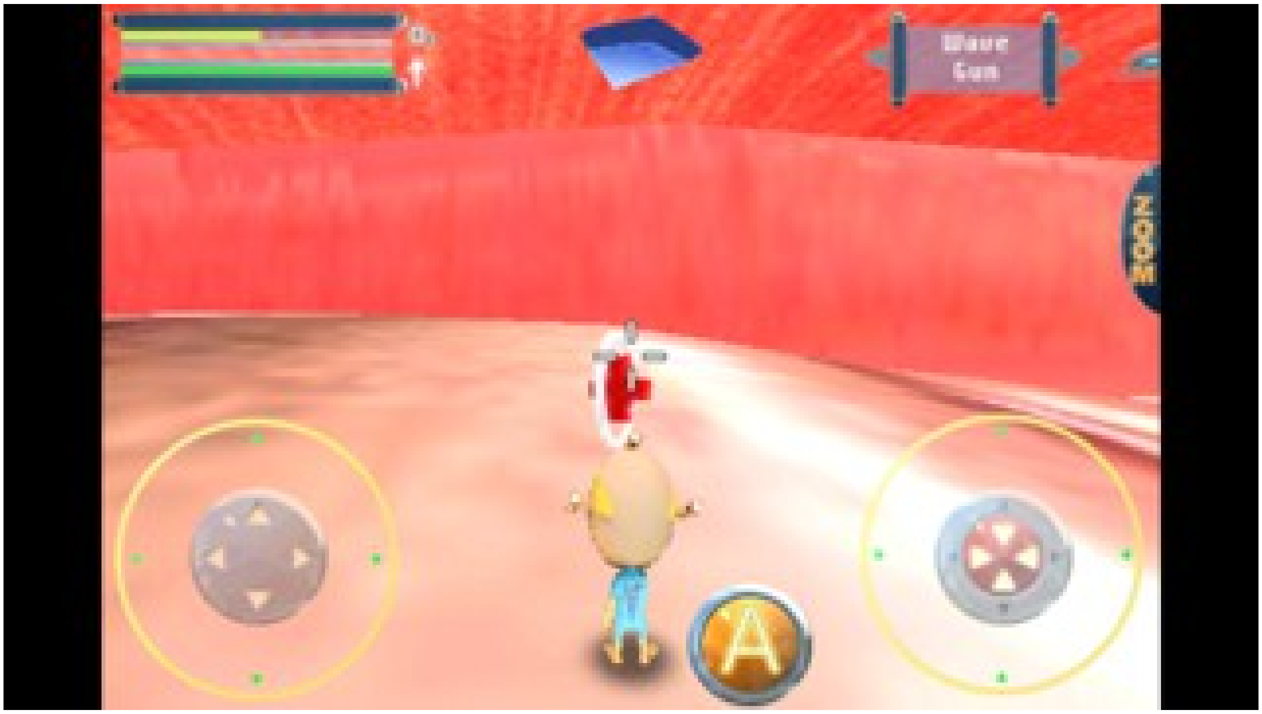

Supplement: Multimedia Appendix 9 [file games_v7i1e11861_app9.pdf]
