# Supplementary material for: Creating a Theoretically Grounded Gaming App to Increase Adherence to Pre-Exposure Prophylaxis: Lessons From the Development of the Viral Combat Mobile Phone Game
Source: JMIR Serious Games. 2019 Mar 27;7(1):e11861. doi: 10.2196/11861 (PMC6456850; doi:10.2196/11861)

Multimedia Appendix 11. Throughout Viral Combat, players engage in various games in order to move on to the next level. In the Condom Sequence game, players must go through the correct sequence of steps for correctly using a condom.

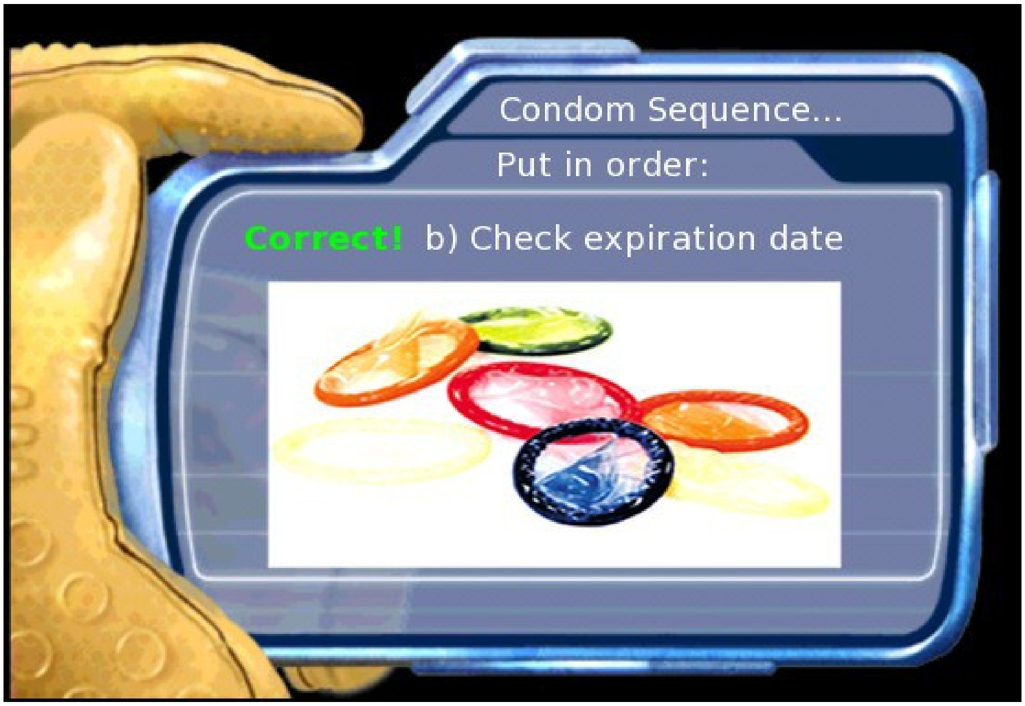

Supplement: Multimedia Appendix 11 [file games_v7i1e11861_app11.pdf]
